# Enterogenic empyema complicating colobronchial fistula: a case report

**DOI:** 10.1186/s13019-020-01211-1

**Published:** 2020-07-23

**Authors:** Yong Yang, Jinghu He, Wei Wang, Ang Li, Gening Jiang, Hantao Wang, Liang Duan

**Affiliations:** 1 Department of Thoracic Surgery, Shanghai Pulmonary Hospital, Tongji University School of Medicine, No. 507 Zhengmin Road, Shanghai, 200433 China; 2 Department of Anorectal Surgery, Changhai Hospital, Second Military Medical University, No. 168 Changhai Road, Shanghai, 200433 China

**Keywords:** General, Empyema, Colobronchial fistula, Lobectomy, Hemicolectomy

## Abstract

**Background:**

Enterogenic empyema is an uncommon complication of colobronchial fistula (CBF). We reported a case of enterogenic empyema patient after surgery for CBF.

**Case presentation:**

A 66-year-old gentleman presented with persistent fever and repeated hemoptysis for 8 months. Computed tomography of the thorax confirmed the presence of a consolidation mass located in the right middle lobe and an air space near the right rib angle. During exploration, CBF was found. The patient underwent right middle and lower lobectomy together with closure of colonic and diaphragmatic perforation. The colon closure and diaphragm closure ruptured after surgery, leading to enterogenic empyema. Adequate drainage, sustained high protein diet, and antibiotic treatment eventually resulted in full recovery.

**Conclusion:**

This is the first report of enterogenic empyema complicating CBF.

## Introduction

The fistula between the respiratory and digestive systems is uncommon and needs to be diagnosed in time because of the severe clinical outcome. The commonest fistula between the respiratory system and digestive tract is tracheal esophageal fistula. Colobronchial fistula (CBF) is rare [1]. There were only 37 cases reported in the past. CBF’s generally present with complicated clinical features and the diagnosis is often delayed. Usually, it is the complication of previous surgery resulting in the connection between the chest cavity and the digestive tract. Systemic investigation to improve the diagnosis and treatment strategy is needed to manage this complicated problem.

In this study, we report an enterogenic empyema complicating surgery to treat a CBF which presented as a lung abscess.

## Case presentation

A 66-year-old man was admitted to our hospital for repeated hemoptysis accompanied by fever for 8 months. A large amount of bloody purulent sputum was coughed out at the beginning, followed by brown pus. The largest amount was about 100 ml. He denied chest pain or dyspnea. The patient took amoxicillin and oral hemostatic drug and the symptoms were relieved. However, the symptoms recurred and gradually worsened. He was admitted to another hospital and received antibiotic therapy with levofloxacin and imipenem. His relevant past medical history included cholecystectomy for gallstone 5 years ago.

**Fig. 1:**
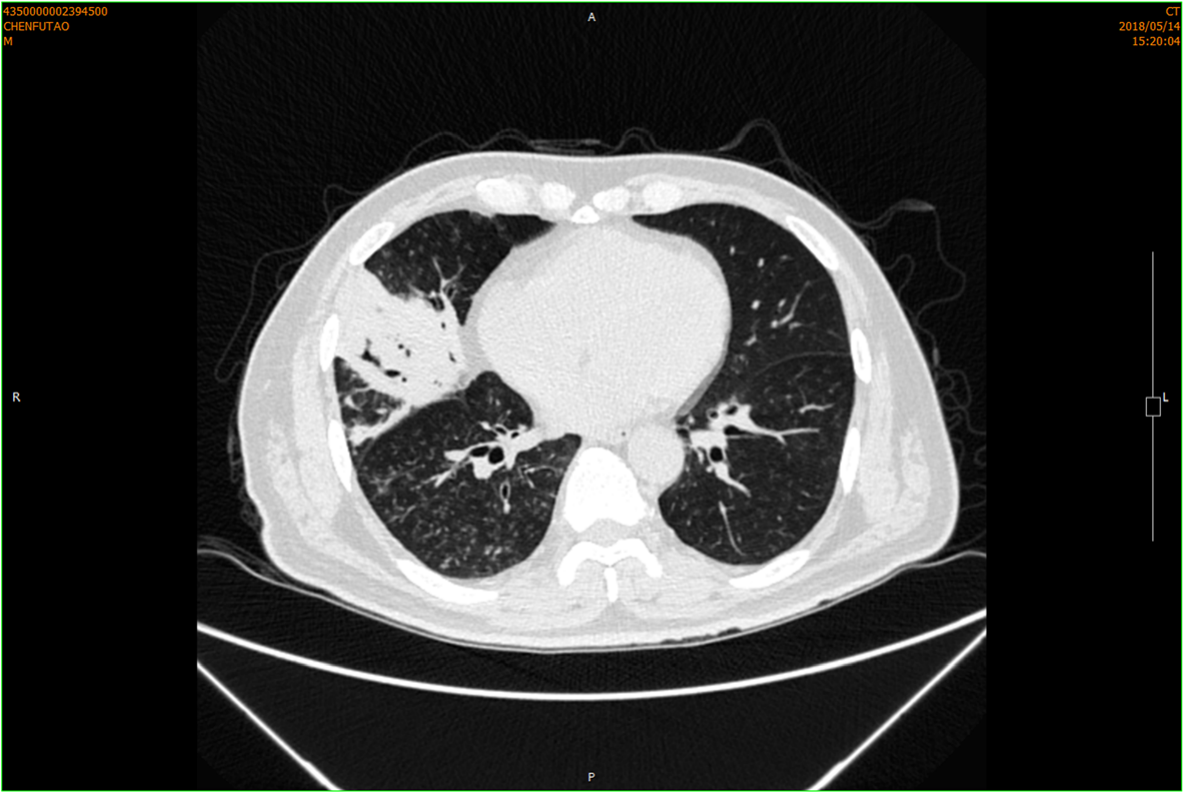
Chest CT scan of the patient on first admission. It was showed a consolidative mass located in the right middle lobe, together with scattered inflammation in the bilateral lower lobes and mediastinal lymphadenopathy

**Fig. 2:**
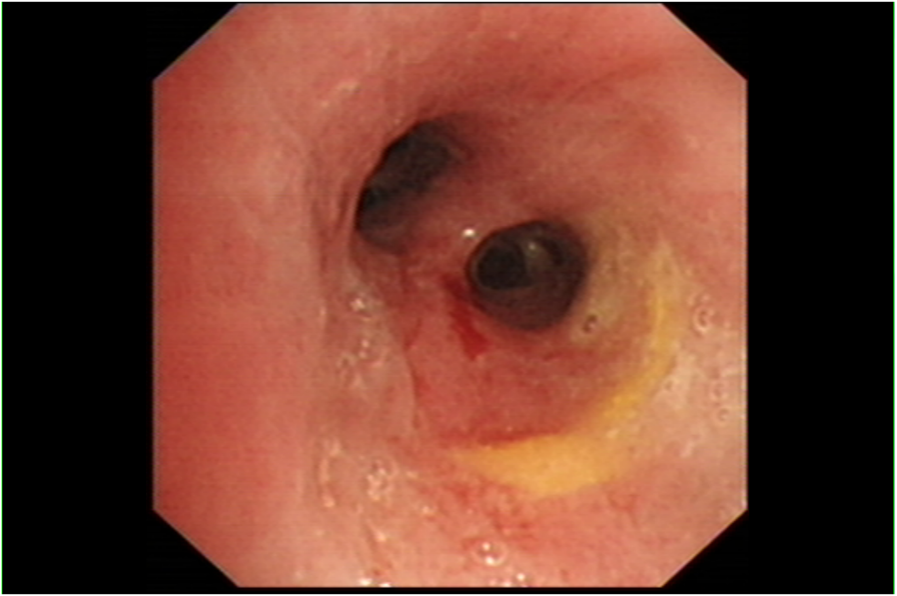
Bronchoscopy examination of the patient on first admission. It was demonstrated no evidence of tumor or tuberculosis

On admission, he had a temperature of 37.0 °C, heart rate of 80 beats per minute, blood pressure of 120/70 mmHg, and an oxygen saturation of 95% on room air. Physical examination revealed no positive sign. Chest CT scan demonstrated a consolidative mass located in the right middle lobe, together with scattered inflammation in the bilateral lower lobes and mediastinal lymphadenopathy (Fig. 1). The patient received ceftriaxone and moxifloxacin intravenously with no improvement. Bronchoscopy exhibited no evidence of tumor or tuberculosis (Fig. 2). Percutaneous lung puncture found inflammatory and tissue cells, and puncture fluid grew *Escherichia coli*. Pulmonary abscess was diagnosed and the antobiotic was changed to biapenem according to the drug sensitivity result (Table 1). The condition improved and the patient was discharged.

**Table 1:** Drug sensitive test of lung puncture sample

Sample type	Test result	Antibiotics	Detection	Drug sensitivity
Puncture fluid	*Escherichia coli*	Amoxicillin clavulanic acid	S	8
		Amikacin	S	<=2
		Ceftazidime	R	> = 64
		Cefepime	R	> = 32
		Cefoperazone sulbactam	I	32
		Ceftriaxone	R	> = 64
		Cefuroxime axetil	R	> = 64
		Cefuroxime	R	> = 64
		ESBL test	P	Pos
		Ertapenem	S	0.25
		Cefoxitin	R	32
		Imipenem	S	0.5
		Levofloxacin	R	> = 8
		Piperacillin tazobactam	S	16
		Compound sulfamethoxazole	R	> = 320
		Tigecycline	S	2

However, the patient presented with hemoptysis again 2 months later but he refused surgery. So, he received right middle lobe arterial embolization. Digital substraction angiography showed a right bronchus common bronchial artery (canal-like expansion of right bronchial artery and right middle lobe pulmonary artery), a left and right common bronchial artery, and abnormal right inferior phrenic artery (tumor-like expansion at the traffic of right inferior phrenic artery and right middle lobe pulmonary artery).

**Fig. 3:**
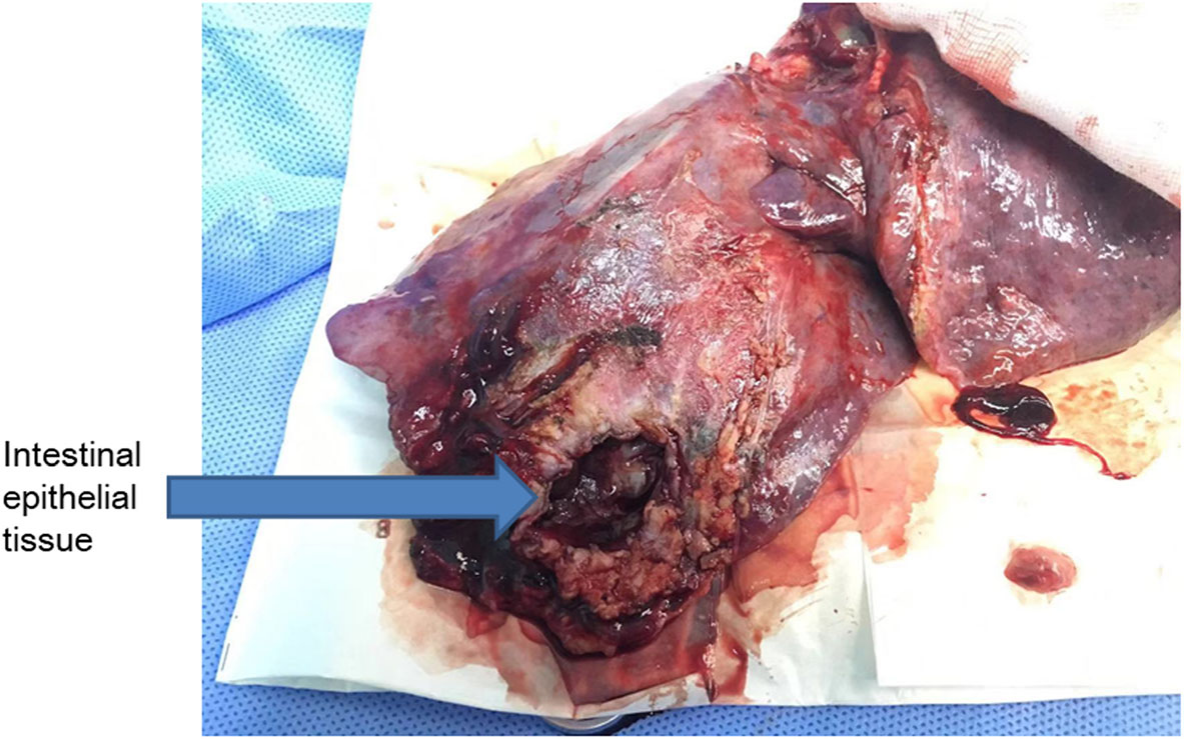
Intraoperative gross specimen of the right middle and lower lobes. Intestinal epithelial tissue was found in the middle lobe

**Fig. 4:**
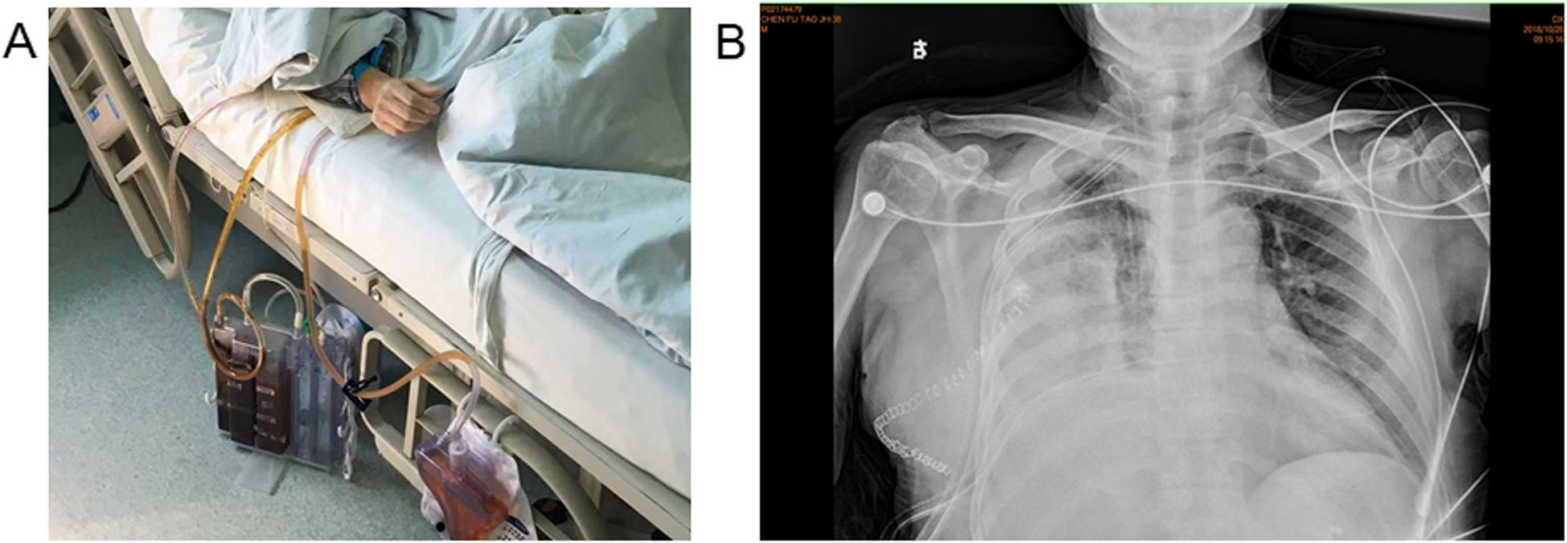
Postoperative empyema. **a** turbid stool like fluid drained out from the chest tube. **b** chest X-ray exhibited decreased transparency in the right chest cavity

**Fig. 5:**
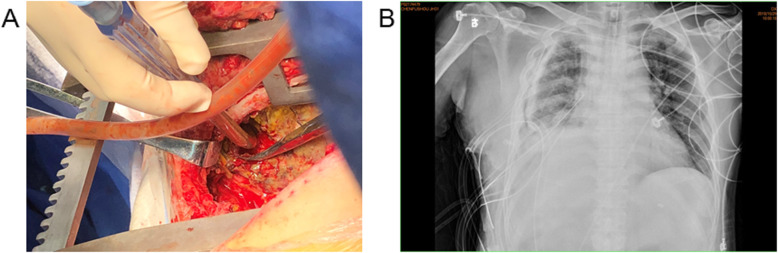
Purulent thoracic clear drainage. **a** intraoperative observation of the diaphragmatic hole connected to the colon. **b** postoperative chest X-ray revealed re-expansion of the lung

Three months later, the patient suffered from hemoptysis again with about 1000 ml a day. Since conservative treatment failed, the patient agreed to right middle and lower lobectomy by open surgery. During the operation, the middle lobe was found to adhere to the diaphragm. However, when the adhesion was removed, a hole was observed on the diaphragm and colonic perforation was seen. The colon and the diaphragm were closed by suturing separately. Pathology demonstrated bronchogenic cysts with epithelial squamous hyperplasia. In addition, intestinal epithelium was found on the adhesion of middle lobe with diaphragm (Fig. 3). Three days after operation, turbid stool like fluid drained out from the chest tube (Fig. 4), and the patient developed continuous fever and increased white blood cell. Enterogenic empyema was diagnosed and emergent surgery found the diaphragm repair ruptured. Considering the pus was derived from the colon, a jejunostomy and empyema drainage was performed. The pus in the chest cavity was removed and a drainage tube was put into the colon from the chest cavity (Fig. 5). Blood culture grew *Staphylococcus aureus*, and he received imipenem and piperacillin/tazobartan alternately upon drug sensitive results (Table 2). The right lung re-expanded after surgery and no drainage came out from the colon tube.

**Table 2:** Drug sensitive test of blood culture

Sample type	Test result	Antibiotics	Detection	Drug sensitivity
Blood	*Staphylococcus aureus*	Vancomycin	S	<=0.5
		Linezolid	S	1
		Moxifloxacin	R	> = 8
		Compound sulfamethoxazole	S	<=10
		Cefoxitin screening	P	Pos
		Gentamicin	R	> = 16
		Levofloxacin	R	> = 8
		Clindamycin	R	> = 4
		Daptomycin	S	0.5
		Rifampin	S	<=0.5
		Koalaranin	S	2
		Ceftolin	S	1
		Clindamycin-induced drug resistance	N	Neg
		Penicillin	R	> = 0.5
		Oxacillin	R	> = 4
		Ergomycin	R	> = 8

**Fig. 6:**
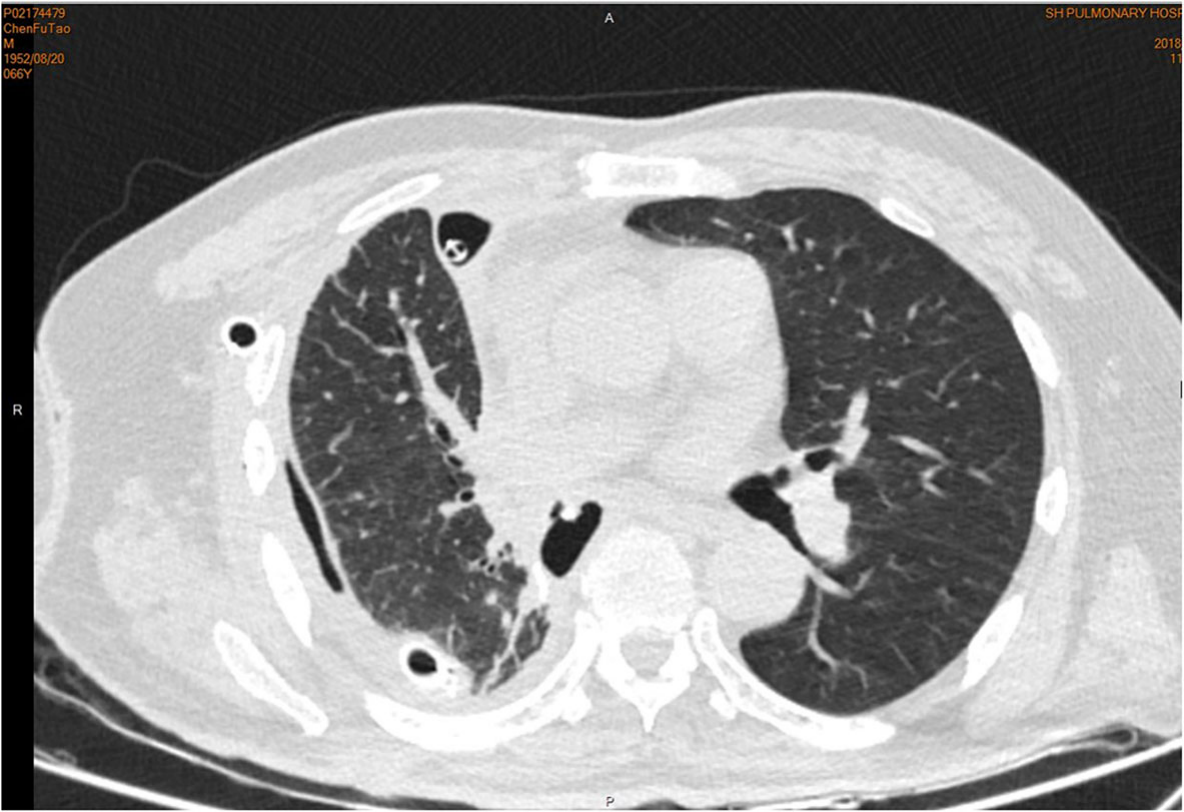
Chest CT scan on the 21th day after second operation. CT scan exhibited BPF on the right intermediate bronchus

Three weeks later, there was air leak from the chest tube again and CT scan showed bronchopleural fistula (BPF) at the bronchial stump (Fig. 6). A rib bed drainage was performed since the visceral pleura was adhered to the lung tissue. Together with large amount of protein intake, the BPF closed in 3 months and all the chest tubes were removed.

**Fig. 7:**
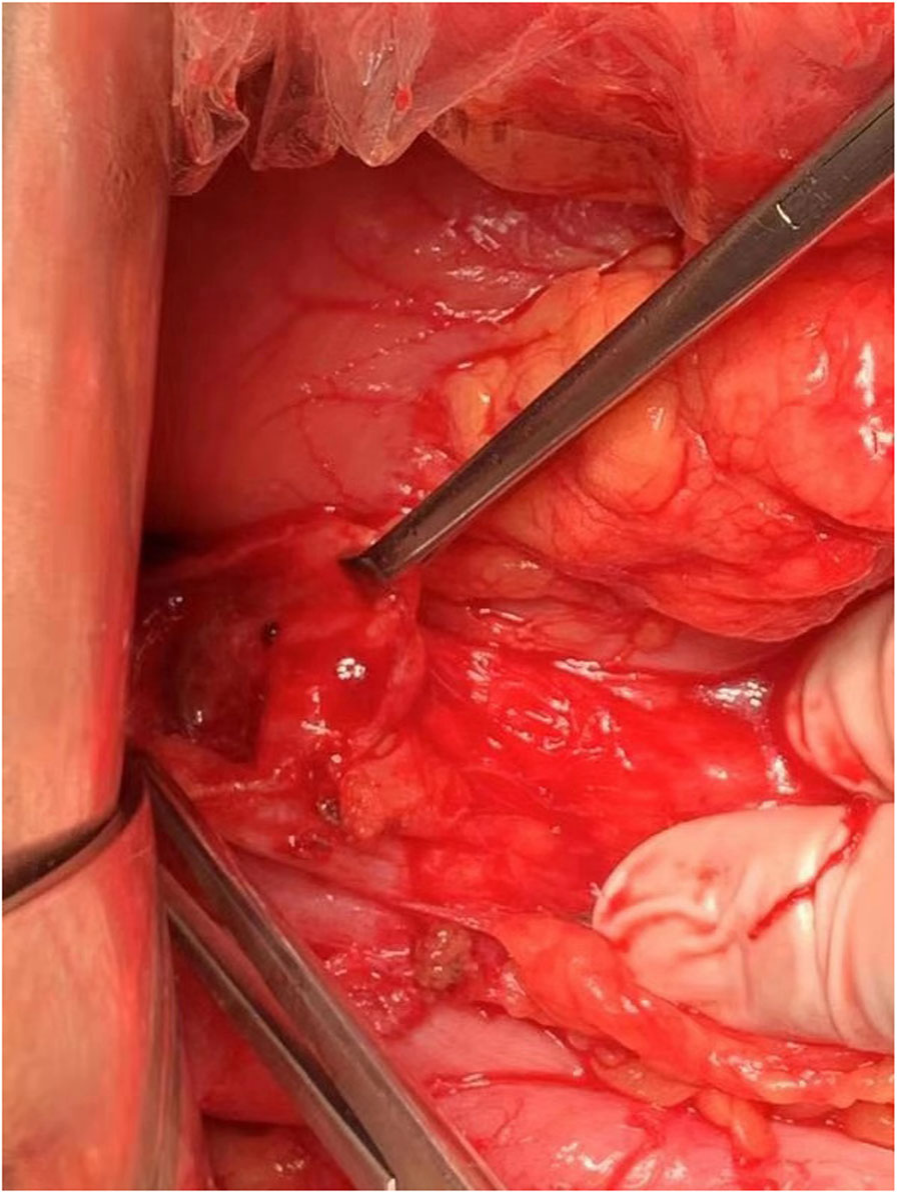
Intraoperative observation of the fistula on the colon. Nine months later, the patient received right hemicolectomy and closure of ileostomy and doing well during follow-up

Nine months after the first operation, enteroscopy demonstrated disuse colitis without other digestive tract pathology. The patient underwent right hemicolectomy and closure of ileostomy (Fig. 7). The pathology reported chronic inflammation without evidence of Crohn’s disease. He was doing well when he was seen in clinic 2 months after the last surgery.

## Conclusion

CBF is an uncommon problem with complicated etiology and clinical presentations. Inappropriate treatment of CBF may result in severe complications. Here, we report the first case of enterogenic empyema complicating a CBF that presented as lung abscess. We hope to better understand this disease and prevent the related complications.

Any disease that can cause the direct or indirect connection between lung tissue and colon may induce CBF. Zhao et al. classified CBF into four types according to the etiology and anatomy [1]. Among them, type I was most common that was CBF secondary to the adhesion among colon, diaphragm, and lung. Both the colon and lung directly adhere to the diaphragm and the fistula forms between colon and lung through the diaphragm. It may be caused by Crohn’s disease, pulmonary infection or abscess, iatrogenic intraperitoneal adhesions, colonic cancer invasion, or pulmonary tuberculosis [2–6]. In this case, the patient suffered from sustained hemoptysis and fever, while the infection was located in the middle lobe, which was near the diaphragm. In general, left sided CBF is more common because of the existence of liver in the right side. However, right side CBF still exists in 20% [7]. Right sided surgery, such as hepatic resection, right nephrectomy, and right diaphragmatic surgery may increase the risk of right sided CBF. Our patient had history of cholecystectomy 5 years before, and maybe it contributed to the cause of his right sided CBF.

CBF can produce both respiratory system and digestive symptoms, of which the respiratory symptoms, such as cough, chest pain, dyspnea, and hemoptysis, are more common [8, 9]. Our patient showed no digestive symptom before operation, further confirming this phenomenon. The typical symptom for CBF is productive cough with foul smelling sputum [10]. Our patient exhibited hemoptysis, while the sputum maroon in color, indicating the sputum may be mixed with stool. However, we did not test the composition of the sputum.

The diagnosis of CBF is not easy clinically because of the complicated pathogenesis and presentations. Since CT scan was widely adopted to observe the pulmonary infection status, it also can be used to visualize the fistula. From the digestive system perspective, flexible colonoscopy is another valuable diagnostic method. A combination of radiological and endoscopic evaluation may obtain a confirmation of CBF. In our patient, CT scan showed an air space near the right rib angle, suspecting the existence of CBF. Meanwhile, sputum culture with positive intestinal flora, such as *Escbericbia coli*, increased the suspicion of CBF [11]. This was reinforced by the finding of *Escbericbia coli* in the culture of pulmonary puncture sample.

The treatment of CBF is difficult and surgery may be the only choice [12]. Sustained infection and digestive problem result in an emaciated. Thus, antibiotic treatment and total parenteral nutrition are essential to improve the patient’s condition for surgical intervention. In this study, our patient received various types of antibiotics resulting in drug resistance. While he was worried about surgery, large amount of oral protein intake was adopted to improve the nutritional status as no digestive symptom was observed. For surgical strategy to manage CBF, the involved colon and lung tissue need to be resected and reconstructed at the same time. The lung and colon should be resected as little as possible for benign CBF, whereas radical resection is needed for malignant CBF. In our case, despite being of benign etiology, the patient received bi-lobectomy because of sustained severe infection and the worry of BPF. The colonic and diaphragmatic openings were closed from the chest cavity simultaneously. Unfortunately, the colonic and diaphragmic both ruptured after operation, leading to enterogenic empyema. It has been suggested that the ruptured colon cannot be sutured in the same period with fistula resection because of the sustained infection. On the other hand, the patient did not show any symptom of peritonitis after empyema, indicating that the enterocoelia may be also adhered, which may be caused by cholecystectomy.

Despite rapid progress in medicine, the mortality of CBF has not decreased significantly [13]. Our patient also developed BPF but he recovered upon adequate drainage, sustained high protein diet, and antibiotic treatment, confirming an effective therapy. The patient finally was cured after reconstructing the digestive tract.

In conclusion, CBF is a rare disease with complicated clinical presentations. It may induce enterogenic empyema after one stage thoracic surgical repair. Adequate drainage, high protein diet, and antibiotic treatment are good strategy for its therapy.

## Data Availability

The datasets analyzed during the current study was available from the corresponding author on reasonable request.

## Abbreviations

CBF: colobronchial fistula
BPF: bronchopleural fistula
CT: computed topography
